# Multi-Class CNN for Classification of Multispectral and Autofluorescence Skin Lesion Clinical Images

**DOI:** 10.3390/jcm11102833

**Published:** 2022-05-17

**Authors:** Ilze Lihacova, Andrey Bondarenko, Yuriy Chizhov, Dilshat Uteshev, Dmitrijs Bliznuks, Norbert Kiss, Alexey Lihachev

**Affiliations:** 1 Institute of Atomic Physics and Spectroscopy, University of Latvia, 1004 Riga, Latvia; aleksejs.lihacovs@lu.lv; 2 Faculty of Computer Science and Information Technology, Riga Technical University, 1048 Riga, Latvia; andrejs.bondarenko@rtu.com (A.B.); jurijs.cizovs@rtu.com (Y.C.); dilshat90@gmail.com (D.U.); dmitrijs.bliznuks@rtu.lv (D.B.); 3 Department of Dermatology, Venerology and Dermatooncology, Semmelweis University, 1085 Budapest, Hungary; kiss.norbert@med.semmelweis-univ.hu

**Keywords:** multispectral reflectance imaging, autofluorescence imaging, convolution neural network, skin lesion diagnostics

## Abstract

In this work, we propose to use an artificial neural network to classify limited data of clinical multispectral and autofluorescence images of skin lesions. Although the amount of data is limited, the deep convolutional neural network classification of skin lesions using a multi-modal image set is studied and proposed for the first time. The unique dataset consists of spectral reflectance images acquired under 526 nm, 663 nm, 964 nm, and autofluorescence images under 405 nm LED excitation. The augmentation algorithm was applied for multi-modal clinical images of different skin lesion groups to expand the training datasets. It was concluded from saliency maps that the classification performed by the convolutional neural network is based on the distribution of the major skin chromophores and endogenous fluorophores. The resulting classification confusion matrices, as well as the performance of trained neural networks, have been investigated and discussed.

## 1. Introduction

### 1.1. Prevalence of Melanoma and Present Diagnostic Approach

Melanoma represents the most aggressive and lethal form of skin cancer. The incidence of melanoma has shown a continuous and dramatic increase over the last several decades, rendering it a significant health burden [1]. While 13 new melanoma cases are diagnosed annually per 100,000 people in Europe, wide variation is seen among different countries, with a higher incidence in Scandinavian countries, the United Kingdom, and Switzerland [2]. The highest incidence rates are reported in New Zealand and Australia, with 50 and 48 per 100,000 people, respectively [2]. The risk of melanoma increases with age. In Caucasians, the overall lifetime risk of developing melanoma is about 2.4% [2]. In the early stages, melanoma can be successfully treated with surgical excision. However, the prognosis is inferior once it metastasizes, and survival rates are considerably lower. Thus, early detection would be crucial improving patient outcomes and reducing mortality [3]. The 2019 update of the European consensus-based guideline for melanoma recommends using dermoscopy for the clinical assessment of skin lesions to diagnose melanoma [4]. However, it notes that dermoscopy requires training and expertise and is not always available, due to a lack of access to dermatologists [4].

### 1.2. An Overview of the Applications of Artificial Neural Networks in the Classification of Skin Lesions

The most promising approaches for image processing are methods that use deep learning (DL) and convolutional neural networks (CNN). Recent studies show that deep neural networks are capable of the differentiation of RGB images of melanomas and nevi with greater sensitivity (82.3%) and specificity (77.9%) than experienced specialists (sensitivity in the range 58–73% and specificity in the range 53–69%) [5]. These results were obtained using the International Skin Imaging Collaboration (ISIC) [6] archive of skin lesion dermoscopic images under white light illumination. In another study, it was demonstrated that a CNN trained using dermoscopy images could augment the diagnostic performance of physicians in the detection of acral lentiginous melanoma [7].

Although many studies on the classification of skin lesions with artificial neural networks have been published recently [8,9,10,11,12,13], more and more new articles appear that correct previous mistakes, improve architecture, etc. These publications and projects mainly use the ISIC archive [6]. Examining the ISIC archive images, artifacts such as a ruler placed on the skin’s surface, colored circle markers, gel bubbles, a black frame, and unique signs drawn by doctors were observed. Such artifacts can cause a false correlation effect. For example, the colored circle markers are the elements that appear in approximately two hundred images with melanocytic nevi only. The effect of colored markers on classification accuracy was described in [14] but not investigated in detail. Natural artifacts, such as hair, shadows, unfocused image effects, jewelry, or tattoos, are permissible, as they occur randomly and should not cause false correlations between the feature and class.

One of the shortcomings of studies is that the results are not transparent enough to evaluate the proposed method. Numerous works [15,16,17] use the area under the receiver operating characteristics (AUROC) metric for evaluating trained (not validated) classifier performance. In such a case, a reduction in performance is possible if the classifier is tested on an alternative validation dataset generated to be the opposite-way imbalanced [18]. The AUROC is not suitable for an imbalanced dataset because false-negative and false-positive diagnoses will impact classification results differently. As well as the change in AUROC has little direct clinical meaning for clinicians [19]. An objective representation of the classification is a confusion matrix, from which sensitivity, specificity, F1 score, and other metrics suitable for assessing classifier performance on imbalanced datasets can be calculated for each classification group [20,21].

Some great works suffer from a lack of k-fold cross validation when they train their models [21,22,23]. Very high classification accuracy may not give a reliable result if it was measured for only one but a lucky data split.

Computer-based analyses such as CNN on RGB skin lesion images under white light illumination have been extensively studied. Although the results are ambiguously explained and interpreted, they have reached an upper limit in their performance [24]. It can mainly be explained by the properties of RGB images, which in generally represent superficial information about lesion texture, color, and shape. RGB images are captured by the CCD matrix (limited by the infrared cut-off filters) under broadband white light illumination. Moreover, the sensitivity curves of the individual R, G, and B channels integrated into the CCD matrix overlap leading to the low spectral resolution of the system. The constructive properties of conventional RGB image acquisition have severe limitations for the selective visualization of tissue chromophores determined by absorption spectra. On the other hand, the spectrally resolved imaging modalities such as multispectral reflectance (MSR) and autofluorescence (AF) may further significantly improve the sensitivity and specificity of skin lesions classification.

### 1.3. Multimodal Spectral Imaging and Its Potential for Deep Learning

The use of deep learning to classify spectrally resolved reflectance and AF clinical images seems to be very promising. Spectral imaging approaches provide spectral information of examined tissues by visualization of tissue chromophores (spectrally resolved reflection imaging) and endogenous fluorophores (filtered AF imaging under selected excitation wavelengths) distribution among the tissue surface. However, due to the lack of available training datasets of spectral images, deep learning approaches’ efficiency is still not presented in the available literature.

Multispectral imaging provides morphological and physiological information of examined lesions, related to the absorption and scattering properties of skin chromophores, such as hemoglobin, melanin and bilirubin content, tissue oxygenation, etc. [25]. MSR imaging for skin cancer diagnostics has been repeatedly demonstrated and clinically proven [26]. Additionally, many of the authors had shown spectral signatures of melanoma in visible and near-infrared regions. Rey-Barroso et al. had demonstrated significant spectral differences between skin melanoma and benign nevi of reflectance spectra at the near-infrared region [27]. Such differences between the reflectance spectra of melanoma and benign nevi have also been studied in our previous research. The most informative wavebands for melanoma diagnostics were defined [28,29]. 

Another imaging modality based on the visualization of endogenous skin fluorophores under UV/VIS excitation could also provide additional information for increasing skin lesions’ diagnostics accuracy. Fluorescence techniques enable the estimation of skin endogenous fluorophores content presented by a mixture of metabolic coenzymes (NAD(P)H and FAD), lipids, structural proteins, vitamins, amino acids, and porphyrins [30]. In malignant tumors, the composition of tissue fluorophores is substantially changed by altered metabolic activity and structural changes [31]. Moreover, in some clinical cases, the AF imaging technique would significantly enhance the primary diagnostics accuracy by differentiating lesions with high AF intensity, such as seborrheic keratosis [32]. It is also known that decreased AF under violet/blue excitation characterizes malignant skin lesions. Recent studies have described notable fluorescence features of pigmented skin lesions under near-infrared excitation. Borisova et al. have demonstrated increased AF intensity of melanocytic lesions in comparison to non-pigmented lesions under 785 nm laser excitation [33]. 

To summarize, the AF and MSR imaging techniques provide additional information beyond conventional clinical photography under white light illumination. Classical processing of MSR and AF image processing followed by subjective interpretation repeatedly has been demonstrated for skin cancer diagnostics. Such “classical” data processing demonstrates a wide range of diagnostics accuracy, mainly dependent on operator experience significantly complicating the routine use in clinical practice. This can be explained by the need to use intensive image processing resources and complex lesion segmentation algorithms that constantly require improvement/tuning due to the wide range of examined lesion types. The use of deep learning for spectroscopic data classification could significantly improve computer-aided diagnostics accuracy, thereby reducing subjective influence on data analysis and interpretation. Moreover, trained CNN could be potentially integrated into the cost-effective spectral imaging devices providing effective screening and diagnostics of skin cancer without any subjective interpretation. An even more valuable effect could be reached in the application of deep learning to estimate the diagnostics efficiency of MSR and AF imaging modalities for their testing and improvement.

However, due to the lack of the available number of clinical cases investigated by spectroscopic modalities, research on optimal CNN training is limited and still not presented in the available literature. The main contribution of this study is to estimate the diagnostics performance of machine learning approaches for the classification of spectral clinical images. The research is aimed at the CNN architecture search to classify limited AF and spectral reflectance data of skin lesions. Within the presented study, we have demonstrated the first results of the classification of MSR and AF clinical images by using a deep learning approach. Obtained CNN training and validation results are presented and discussed below. 

## 2. Materials and Methods

### 2.1. Proposed Approach

In our previous studies, using the optical density images taken at 51 wavelengths, the three most sensitive (540 nm, 650 nm, and 950 nm) were selected according to the optical properties of the skin. Additionally, melanoma differentiation parameter *p’* was developed to distinguish melanoma from nevus with high sensitivity and specificity [28]. Later, discovering that using AF (induced by 405 nm) can distinguish seborrheic keratosis from melanoma [32], the diagnostic method was supplemented with more skin lesion groups [34]. To make the classification more objective and automated, it was decided to create a skin lesion classifier using CNN. First, to distinguish melanoma from other skin lesions, the most sensitive waveband triplets from 51 (450–950 nm) channels were identified [29]. Experiments using InceptionV3, VGG16, and ResNet50 pre-trained networks were performed. InceptionV3 was chosen because of the good balance between the reported classification performance and the complexity. VGG16 and ResNet50 architectures are simpler but faster models. All the mentioned architectures were pre-trained on the ImageNet dataset. Then, the output (their fully connected layers) was dropped entirely and replaced with global average pooling, followed by the one or several fully connected layers. Several options for added “tail” layer structure were tried: presence or absence of batch normalization and dropout layers followed by the obligate sigmoid layer. Binary cross-entropy was used as a loss function in the training procedure. All original filters learned during pre-training were frozen and remained intact during our training procedure. Our training procedure adjusted only the added mentioned layers. Only InceptionV3 architecture showed promising results. Experiments with other pre-trained networks were abandoned. In the case of InceptionV3 additional experiments were conducted. The first 250 layers were frozen, and the rest were trained with the “tail” of the network with fully connected layers. The model with all original frozen weights and two dense layers interconnected with dropout and batch normalization showed the best performance. However, the handmade CNN performance was better.

Using a custom multispectral dataset, a simple six-layer CNN (ConvNet) was compared to a fine-tuned (both fully connected classification layers and CNN filters with varying learning rates) InceptionV3 network. Handmade CNN model had the following structure: input layer accepting 150 × 150 × 3 tensor, followed by four pairs of ReLU convolutional 2D layers with the same kernel size (3 by 3) and with 32, 64, 128, and 128 filters, respectively, followed by 2D max pooling layer with pool size 2 by 2 without padding, all of that followed by flatten layer, then by dense layer with 512 ReLU neurons and concluding sigmoid layer with 1 neuron. The mediocre performance of the simpler VGG-16 and ResNet50 could be explained by the smaller amount, complexity, and hierarchy of their filters; therefore, they were excluded from further experiments. The success of the simple CNN trained from scratch could be explained by the fact that InceptionV3 was trained on feature-rich (texture-rich) RGB images (photos of the surrounding world), and trained RGB filters did not apply to our dataset. It is a known fact that CNNs are relying on textures for successful classification [35]. In our case, we assume that the data contains mainly spectral information, not texture. Having described results, we opted for a training-from-scratch approach and decided to look for an architecture fine-tuned to our small dataset with the help of neural architecture search, which would produce CNN of simpler (in comparison to, e.g., InceptionV3) structure. In addition to CNN, we have conducted 5-fold cross-validation over 5-fold cross-validation of the visual transformer architecture adapted for rather small datasets [36], which showed mean validation F1-score of only 67.64%, compared to 75.38% for best DARTS architecture (experimental setup was the same as for the following experiments and will be described below, the parameters of the ViT was: image_size = 128, patch_size = 16, num_classes = 5, dim = 1024, depth = 6, heads = 16, mlp_dim = 2048, dropout = 0.1, emb_dropout = 0.1, channels = 4). In addition to transformer architecture, we have evaluated current state-of-the-art ConvNext architecture [37] (preliminary experiments using stratified 5-fold cross-validation with default parameters using image size 224 × 224) which produced average (over 5-fold) weighted F1 scores of 0.618 and 0.559 for training and test datasets accordingly. ConvNext saliency maps exploration showed signs of overfitting, as in many cases the model was paying attention to the black markers placed on skin for subsequent images alignment (registration). This seems to result from the high resolution of images and high capacity of the architecture, which led to overfitting. We decided to utilize a differentiable architecture search (DARTS) [38] CNN with only several hidden meta-layers. Network architectures of depths from 1 to 5 meta-layers with inner channels equal to 10, 12, 14, 16, and 18 were searched, trained, and evaluated. 

In the current work, we chose early data aggregation; otherwise, the relationships between signal strength across different channels will be lost (as each channel would be processed separately). As a result, we fused separate single-channel images into four-channel images (3D tensors) that were used to train a classification model.

Currently, three main approaches for deep learning systems training are: manual architecture construction, transfer-learning, and neural architecture search [39,40,41]. We used transfer learning alongside manually created networks to assess the importance of wavebands triplets in previous research. Transfer learning (Inception V3) showed lower performance than manually constructed architectures. However, even manually created models have not been able to attain greater than 50% F1 classification score. All latest state-of-the-art results from the International Conference on Learning Representations (ICLR) image classification competitions were acquired using so-called neural architecture search approaches. Considering the multi-spectrality of our dataset, its small size, and the fact that we are dealing with non-RGB images, we opted for the neural architecture search.

DARTS algorithm was applied to search for the best possible architecture capable of performing skin lesions classification. We hypothesize that simpler Deep Neural Network architecture (compared to existing pre-trained architectures) is required as there is no rich visual diversity within our dataset composed of registered stacks of four images taken at different wavebands. These images mainly do not contain any textures. A combination of absolute pixel intensity values across all available channels can be used for the classification. Any intensity-related augmentation disrupts the relationship between channels (reduces spectral resolution) and causes poor classification performance. In contrast, classification by CNN in regular photos is based mainly on textures [35]. As a result, a five meta-layer neural network architecture was found and trained for five class discrimination, containing only ~32,000 trainable parameters. Models were composed of dilated and separable convolutions. Both are focused on computational cost reduction. Found architectures include many computationally simple 1 × 1 convolutions blocks combined with 3 × 3 and 5 × 5 convolutions and organized in several parallel pipelines merging at different depths. Experiments have shown that 350 training epochs are sufficient, which can be explained by the dataset’s small size. Manually created architectures always delivered a worse training performance due to the higher number of training parameters.

### 2.2. Description of Multispectral Data

Multispectral data of different skin lesion diagnoses were used in this work (breakdown by diagnosis is shown in Table 1). The multispectral image sets were obtained by a previously developed portable multispectral imaging device [42]. The multispectral device was equipped with narrow-band LEDs: 526 nm (green), 663 nm (red), and 964 nm (infrared) to obtain diffuse reflectance images, and 405 nm for the acquisition of filtered (515 nm long-pass filter) skin AF images. To avoid artifacts caused by specular reflection from the skin surface, the polarization of the illuminating light was orthogonal to the orientation of the polarizer located in the camera lens. Images were acquired by an IDS camera. For instance, one dataset that has been analyzed consists of four images: a green (G) channel from an RGB image obtained under 526 nm illumination, a red (R) channel from an image obtained under 663 nm illumination, an R channel from an image obtained under 964 nm illumination and G channel from AF image. All sets of images were registered (aligned) using an optical-flow-based registration routine.

Melanoma-like lesions diagnosis (Table 1) was approved by histological evaluation. Diagnosis of benign skin lesions was approved clinically under the supervision of an experienced dermatologist/dermato-oncologist. The Ethics Committee has approved this study. The research has been conducted in accordance with the Declaration of Helsinki and the Oviedo Convention.

Multispectral data were collected from individuals with Caucasian skin type at the Oncology Center of Latvia (Riga, Latvia) and at Semmelweis University (Budapest, Hungary) under the supervision of medical physicists, dermatologists and dermato-oncologists. 

### 2.3. Data Preprocessing

At first, data were visually analyzed to remove from the dataset incorrectly captured data. These images were observed to have unacceptable artifacts such as severe motion blur or one or more missing channels G, R, IR or AF.

The images were collected from several self-made equipment models and stored in PNG format. Due to equipment that has been redesigned and improved over time, the images have different sizes (960 × 686, 460 × 512, and 640 × 480 pixels). Since DARTS models were trained on Nvidia GeForce 1080 Ti (11Gb GDDR5X), we reduced the original image size to meet memory requirements. The final solution used 4 × 128 × 128 input images. Other tested input image sizes will be discussed in Section 4. To minimize distortion effects, image resolution reduction was performed using bicubic interpolation over a 4 × 4 pixel neighborhood. In the next step, image sets were aligned using the OpenCV OpticalFlow algorithm to compensate for motion artifacts between different channels. Optical Flow uses the shape and position of the marker on the skin (the presence of a marker is crucial as some of the images have a certain degree of blurriness, and no considerable details are present that can be used to acquire multimodal images). As a result, 4 × 128 × 128 tensors were created for the CNN training and validation procedure. 

### 2.4. Data Augmentation

A total of 20,864 (1304 original and 19,560 augmented) multispectral datasets of skin lesions were used. 

As our accumulated dataset is rather small and no such open-source multi-modal data is available to supplement our database, a commonly used image augmentation approach [43] was applied to avoid overfitting and improve the overall performance of the trained CNN. In total, 15 variations of augmented multispectral versions were created for each original 4 × 128 × 128 input, increasing the overall amount of data by 16 times. The augmentation was accomplished using random horizontal/vertical flipping (with a probability 0.5) and random rotation (−90°, +90°, with a probability 0.5) of the original images. In our case, the augmented data was only rotated and mirrored without adding other specific features. Normalization of images or any other color-related augmentations severely reduced the performance. Augmented image sets were used for training, and only original data were used for validation. Additionally, one of the channels—G, R, IR, or AF—was randomly removed with a probability of 0.2 (higher and lower probability reduced the classification performance). The use of this procedure reduced overfitting.

Posed classification problem was aimed at the classification of images as belonging to one of the five following classes:

- Melanoma-like lesions (MLL);
- Pigmented benign lesions (PBL);
- Hyperkeratotic lesions (HKL);
- Non-melanoma skin cancers (NMSC);
- Other benign lesions (OBL).

### 2.5. Validation of Classification Decision via Saliency Map

**Figure 1:**
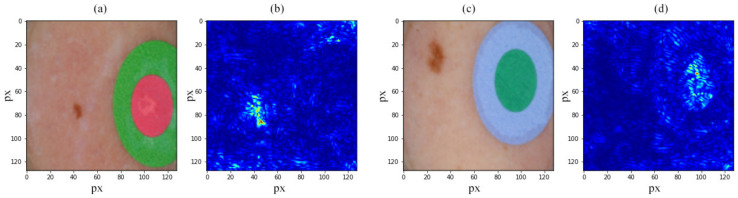
Nevus and marker RGB images from ISIC archive (**a**,**c**) and saliency maps acquired via pre-trained ResNet50 fine-tuned on ISIC dataset showing good inference relying on the lesion (**b**) and inference relying on a marker, which shows model overfitting (**d**).

One method to evaluate the performance of trained neural networks is to investigate the resulting saliency maps [44]. We used saliency maps to validate and understand the classification decision of our DARTS found CNN architectures. Saliency maps of the correctly trained network should focus on the areas of the lesion that contain essential classification features. If the focus is shifted to artifacts (markers, corners, hair, etc.), the neural network should be considered overtrained (i.e., overfitting). The intensities of the saliency map characterize the importance of each pixel across all input image channels with respect to the classification results. For assessment of the mentioned approach, we used the ResNet50 network pre-trained on the ImageNet dataset in a transfer-learning scenario (fine-tuned, i.e., only the “tail” was trained). Figure 1 shows the saliency maps acquired from a neural network fine-tuned on the ISIC dataset. Figure 1b shows a saliency map built for input in Figure 1a. Figure 1b demonstrates that the trained model considers the lesion to perform classification. Figure 1d shows network inference acquired using Figure 1c, which considers the marker to make a classification decision—the unmistakable sign of overfitting. Here, low importance (classification-wise) areas are marked in the dark blue, and high importance areas are marked in red. One way to overcome this problem is data augmentation. In this case, when only some of the lesions in the ISIC archive have such markers (mentioned in Section 1.2), it should not be a rotation but image cropping to remove markers from the dataset. Another approach is the elimination of such data samples from the dataset.

## 3. Results

### 3.1. DARTS Applying for Network Architecture Search

**Figure 2:**
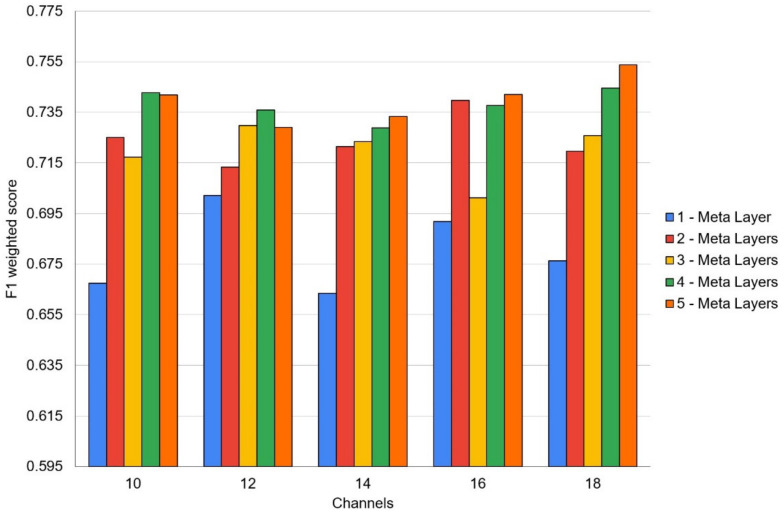
DARTS hyperparameters (layers and inner channels) estimation experiments result of mean F1-weighted validation scores acquired on 5-fold stratified cross-validation executed over 5-fold stratified cross-validation.

Several neural architecture search sessions were performed for networks with different depths ranging from 1 to 5 layers in this work. In the context of the DARTS model, these layers should be considered as meta-layers consisting of several parallel data flows. Hyperparameters influence experiments were conducted using 10, 12, 14, 16 and 18 internal data processing channels used in the DARTS layers. Figure 2 shows the resulting mean F1-weighted validation scores attained during these experiments. The experimental setup is described below.

Each experiment involved five-fold stratified cross-validation running over five-fold stratified cross-validations. The confusion matrices were used for reporting test results using the data acquired from the outer test fold using the best model, which showed the best validation F1 score acquired from the inner validation fold. Hence, five test confusion matrices were combined to present the averaged result over five outer folds tests sets. Among these experiments, 18 internal data processing channels of five meta-layers showed the best validation classification score. In the scope of a single DARTS layer search, we used the following operators: MaxPooling, AveragePooling, SkipConnection, SepConv 3 × 3, SepConv 5 × 5, DilConv 3 × 3, DilConv 5 × 5. SepConv operator is responsible for depthwise separable convolution [45], but DilConv is responsible for dilated convolutions [46]. As a result, five network architectures of varying depth (from one to five hidden meta-layers) trained to classify MLL vs. PBS vs. HKL vs. NMSC vs. OBL were found. In our case, a single layer is not a classical CNN layer containing convolution and pooling layers, but holding several operators combined into a non-trivial pipeline. 

Moreover, these operators were stacked together to form a complex operation part of one or several pathways in the single “layer” of the architected CNN. It would be more appropriate to call these layers meta-layers. Networks used in this work contained one to five such meta-layers. Furthermore, in the final multi-layered networks, even subsequent meta-layers are not the same; they vary in their inner structure.

The use of architecture search tools reduces time costs compared with the manual approach. The time cost depends on the dataset size, architecture complexity, number of folds, and image size and structure. It took ~50 h to process on GeForce 1080Ti GPU, 11Gb video RAM for the four-channel images (128 × 128 pixels), five meta-layers deep network, and five-over-five folds of stratified cross-validation. An asynchronous process was implemented for image loading and preprocessing routines, and neural network training on GPU. The definition of the search space for the architecture (convolutions of size 3 × 3 and 5 × 5) was slightly reduced compared to the original, which used larger convolutions. This was done due to memory limitations that should be satisfied, which is a common problem with all neural architecture search algorithms. From the implementation aspect, the PyTorch DARTS package of the MS NNI framework [47] was used.

### 3.2. Results Using Found Architectures and Trained Networks

**Figure 3:**
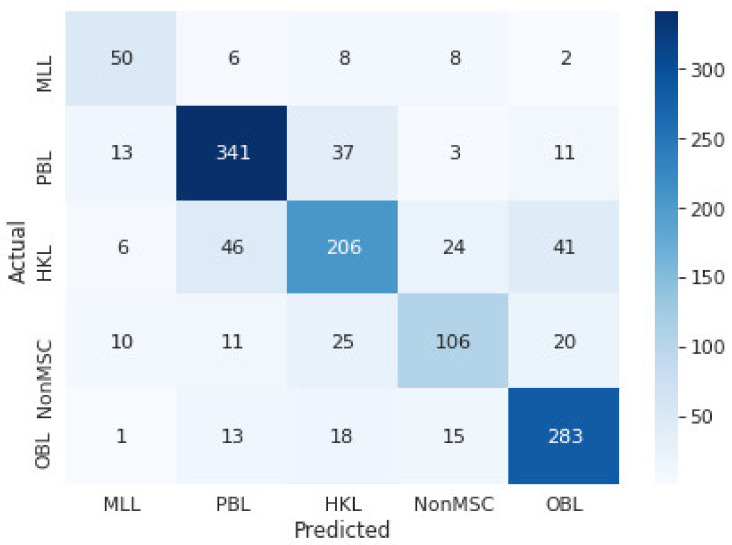
Confusion matrix of the validation set for MLL vs. PBL vs. HKL vs. NMSC vs. OBL.

Since the dataset size is small, the cross-validation over cross-validation technique was used. That allowed using all the available data for training and ensuring that all k-fold trained models were tested on separate datasets. For a more objective assessment of the classification performance of the found architecture and trained deep neural network, the results are presented in the form of confusion matrices obtained as a sum of five-fold stratified cross-validation folds over five-fold stratified cross-validation for the test datasets of the outer folds (Figure 3). For inner cross-validation folds, training for 350 epochs was performed, and simultaneously the best models (showing highest F1-validation score) were saved. Afterward, the best-performing models from the inner folds were selected to be tested on the outer fold test dataset. We are saying models because we have tried both final models trained over 350 epochs and the best model found during training (considering test set performance). In the end, models trained over 350 epochs showed slightly lower levels of overfitting. Figure 3. shows the hyperparameters search results for the combined validation sets confusion matrix for the classification group: MLL vs. PBL vs. HKL vs. NMSC vs. OBL.

Our further goal is to create an artificial neural network that would be suitable for medical practice. A multispectral device with a built-in trained artificial neural network will help inexperienced physicians decide if a patient needs to be referred for further examination. The average F1 test score is 0.749. Sensitivities and specificities for each specific class can be seen in Table 2. In particular, the specificity tests for all examined groups demonstrated values above 0.9. The highest sensitivity values are obtained for groups of MLL (0.72), BPL (0.83), and OBL (0.84). Obviously, the sensitivity value for melanoma discrimination from benign pigmented lesions shall be higher to exclude missed melanoma cases. However, diagnostics values obtained by the classification of spectral images by CNN are suitable for developing portable devices that provide additional diagnostics support in clinical practice. Such devices will be designed to provide convenience and ease of use even for inexperienced professionals, and the devices should include as many different groups of skin formations as possible that can be confused with each other. In this case, we choose to use a classifier with five groups, which we plan to retrain over-time on the newly acquired skin formation data.

### 3.3. Prediction Validation Using Saliency Maps

We extracted saliency maps and verified if classification results were correct and appropriate to test if our CNN model is trained correctly. In the case of incorrect training (e.g., overfitting), the neural network highlights the attention to the marker or other artifact-forming regions such as hair, edges, shadows, etc. In our dataset, the black marker was added to each image that is used to align the image set. The marker retains the same shape in all images, with minor variations due to the skin’s curvature and the angle at which it is recorded. Since the marker is added to all lesion types and is not correlated with diagnosis, saliency maps should exclude the marker from classification.

**Figure 4:**
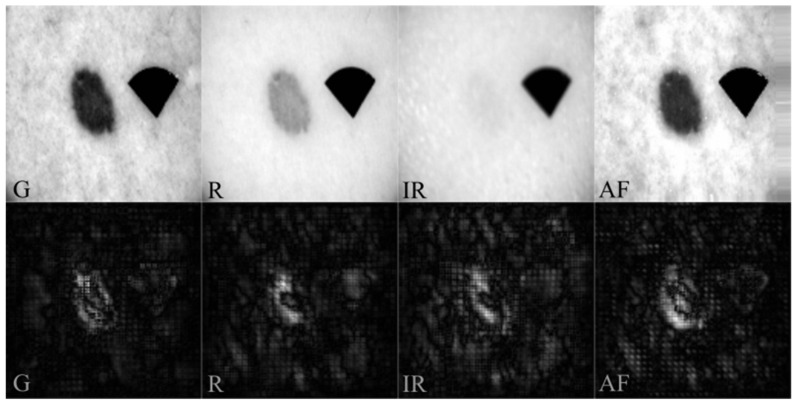
Input multispectral G, R, IR, and AF images (**top** row) and corresponding saliency maps (**bottom** row). PBL input images were recognized as PBL (classification of MLL vs. PBL vs. HKL vs. NMSC vs. OBL).

Figure 4 displays representative examples of input datasets and the resulting saliency maps for each channel. For PBL (Figure 4), almost the entire lesion shows high intensity in the saliency maps, containing asymmetrically distributed pigmentation with a uniform structure and regular borders. Saliency maps showed an increased number of bright pixels in regions with high blood (540 nm), melanin (630 nm), and water (950 nm) optical absorption and variations of endogenous skin fluorophores content under violet excitation. Accordingly, including near-IR and AF images for CNN training are reasonable and appropriate.

## 4. Summary and Discussion

The objective estimation of multispectral computer-aided diagnostics (CAD) accuracy is a complicated scientific and practical task that is challenging to solve. The physician’s role in influencing the choice of the region of interest and patient selection for the examination has a significant impact on resulting diagnostics sensitivity and specificity. Moreover, most CAD studies are performed in preselected patients’ groups with high melanoma prevalence, which can also affect the resulting sensitivity parameters. According to available literature data, the CAD of multispectral imaging systems (in total, five different spectral imaging systems were tested in 18 studies) have provided an average sensitivity of 92.9% (83.7% to 97.1%) and specificity of 43.6% (24.8% to 64.5%) for melanoma discrimination from atypical intraepidermal melanocytic variants [26]. In these studies, 2401 skin lesions, including 286 melanomas, were examined. CAD diagnostics results demonstrate a high sensitivity for detecting invasive melanoma and atypical intraepidermal melanocytic variants. However, specificity parameters of the multispectral imaging systems deliver low values and vary within different studies [26]. 

In the presented research, we have processed 20,864 (1304 original and 19,560 augmented) MSR and AF sets of clinical cases. The confusion matrices and resulting diagnostic parameters such as sensitivity and specificity were obtained for the classification of MLL vs. PBL vs. HKL vs. NMSC vs. OBL. As seen, the CNN system shows high specificity values for melanoma-like lesions (0.97) and non-melanoma skin cancer (0.95). Obtained specificity values demonstrated significantly higher values than values obtained by CAD systems (43.6%). However, the sensitivity of the CNN classification of spectral images showed lower values (MLL 0.72 and NonMSC 0.57) compared to CAD (92.9%). Undoubtedly, future research should be addressed to enhance sensitivity to avoid missing cancerous cases in routine screening. Future expansion of the dataset, including more clinical cases, seems reasonable to develop even more precise classification models and reach higher sensitivity rates. However, in the presented research, including more skin lesion groups in the classification model, the diagnostics parameters decreased, which is acceptable because most lesions are rather poorly represented (less than 100 samples overall). This can be explained by the fact that improving the CNN skin lesion classifier requires not only increasing the number of skin lesion groups, but it is necessary accumulating a significant amount of data in each skin lesion group. Increasing the size of the dataset always positively influences the generalization abilities of classifiers and serves as a regulator. Suppose the increase in dataset size is achieved by the introduction of other classes (e.g., new skin lesion groups). In that case, we are not exposing the network to diversified samples of a single class but are adding samples of new classes. With a high probability, such addition of data will reduce the performance of the classifier. We hypothesize that training a classification model for discriminating specific lesion types (by diagnosis) instead of groups of lesions should improve the classification performance. Therefore, the constant expansion of the dataset is crucial in developing even more accurate classification models.

Overall DARTS algorithm allowed us to find neural network architectures capable of performing classification on limited data of spectrally resolved reflectance and AF clinical images of skin lesions. Assessment of classification performance on validation sets showed signs of overfitting, despite various regularization tricks, such as random channel dropout, rotation, and slight regularization of weights within the optimization algorithm. Despite that, the validation performance was at a satisfactory level.

Another important issue affecting the classification performance of CNN, and one which could be addressed, is image spatial resolution and clarity. In the presented study, the imaging setup is equipped with cross-polarized filters enabling capturing diffuse reflectance images from deeper skin layers, avoiding specular reflection from the skin surface. Thus, the quality or clarity of the acquired multispectral and AF images mainly depends on incident light penetration depth to the tissue. The shallowest light penetration depth enables the highest clarity of the images. While the deeper penetration depth of the incident light makes diffuse reflectance images significantly blurred. In our experiments, images with the highest clarity are AF images under violet, excitation, and spectral reflectance images under green illumination, since the penetration depth of violet and green light is significantly smaller than red and infrared. Accordingly, the red and infrared images are much more blurred. The overall number and pixel intensity depth are similar for all illumination’s wavelengths and detection. The impact of spatial resolution and clarity parameters on the CNN classification performance should be studied in more detail. Optimal image resolution was observed and applied in the experiments. Found and trained on NN, the classifier model architecture used images with a resolution of 128 × 128 pixels. Experimental testing on a slightly higher resolution (192 × 192 pixels) and lower resolution (96 × 96 pixels) images resulted in a lower training and validation classification performance. We hypothesize that more refined and more diverse morphological information, such as vascularization, melanin network, etc., is visible at a higher resolution. Most probably, the images with the highest clarity (green and AF) become over-dominant to the red and infrared images, leading to an upset of cross-correlation weights between the whole image set. Such features may be present in several skin lesion diagnoses from different established skin lesion groups, resulting in reduced classification performance. Such information, combined with a massive amount of data, could significantly improve the classification of skin lesions at the level of diagnosis. Still, higher resolution reduced the CNN classifier performance in our case (with a small amount of data in each skin lesion group and some slightly blurred data samples). On the other hand, too much morphological information was lost in too low resolution (96 × 96 pixels) images, which leads to the conclusion that the found architectures consider not only the spectral information between the channels but also the shape (e.g., irregular borders) and size.

## 5. Conclusions

The use of deep learning for the fully automatic classification of multimodal (MSR and AF) clinical images raises a well-grounded practical interest for dermatologists and technology developers. However, the diagnostic performance of deep learning approaches for the classification of multi-modal clinical images has not been fully studied and evaluated, due to the lack of available large training datasets. Additionally, available literature frequently mentions the potential of deep learning for the classification of multispectral images and lacks results of complementary research. Our unique research is one of the first attempts to classify multimodal clinical images by using CNN. We proposed using spectrally resolved reflectance and AF images for CNN training and classification within the presented research. Obtained saliency maps demonstrated high weights on CNN decision making attributed to the skin’s significant chromophores (hemoglobin, melanin, water) and endogenous skin fluorophores. Overall, the presented approach for CNN construction is suitable for classification and could be helpful for various sets of MSR and AF images. 

Based on the presented study, we conclude that multimodal (with spectral resolution) datasets (small or medium-sized) that do not contain a large variety of textures should not be approached using models pre-trained on feature-rich datasets. Our experiments have shown poor performance of VGG16, ResNet50 and InceptionV3 architectures, which a simple handcrafted CNN outperformed. We hypothesize that in the described scenario, a custom trained-from-scratch architecture should be favored over the transfer-learning approach using classification models trained on texture-rich datasets.

Further research would require exploring deeper architectures with an expanded dataset. Such studies would require a more significant amount of GPU RAM (we had 11GB) and could potentially lead to the learning of some texture-specific filters by a trained CNN, which may be important in the process of classifying specific skin lesions.

For production purposes, we expect to use the entire dataset for network training. A bigger dataset will improve generalization capabilities. Continuing this study, the dataset is continuously updated with new data samples obtained in hospitals in Latvia and Hungary. 

## Figures and Tables

**Table 1:** Distribution of skin lesion groups and the size of multispectral image sets.

Lesion Group	Seq. No.	Lesion Diagnosis	Size of Multispectral Datasets (N)	Size of Augmented Multispectral Datasets (N_aug_)	The Total Size of Multispectral Datasets (N_tot_)
Melanoma-like lesions, **MLL**	1	Malignant melanoma, **MM** (C43)	70	1050	1120
	2	Lentigo maligna (D03.9)	4	60	64
Pigmented benign lesions, **PBL**	3	Melanocytic nevus, **MN** (D22)	394	5910	6304
	4	Ephelides (L81.2)	1	15	16
	5	Lentigo solaris (L81.4)	7	105	112
	6	Congenital nevus (Q82.5)	3	45	48
Hyperkeratotic lesions, **HKL**	7	Seborrheic dermatitis (L21)	3	45	48
	8	Actinic keratosis (L57 + L57.0)	12	180	192
	9	Seborrheic keratosis (L82)	129	1935	2064
	10	Hyperkeratosis (L85)	89	1335	1424
	11	Cornu Cutaneum (L85.5)	2	30	32
	12	Anogenital warts (A63)	6	90	96
	13	Ichthyosis vulgaris (Q80)	6	90	96
	14	Papilloma (B07)	41	615	656
	15	Skin changes due to chronic exposure to nonionizing radiation (L57.9)	35	525	560
Non-melanoma skin cancer, **NMSC**	16	Basal cell carcinoma (C44)	165	2475	2640
	17	Carcinoma in situ (D09)	6	90	96
	18	Keratoacanthoma (L85.8)	1	15	16
Other benign lesions, **OBL**	19	Hemangioma (D18)	36	540	576
	20	Myxoma (D21.9)	5	75	80
	21	Granuloma annulare (L92)	6	90	96
	22	Calcinosis cutis (L94.2 + PXE)	59	885	944
	23	Other specified disorders of the skin and subcutaneous tissue (L98.8 + L98.9)	48	720	768
	24	Sarcoidosis (D86.3)	5	75	80
	25	Healthy skin (ada)	171	2565	2736
Total			**1304**	**19,560**	**20,864**

**Table 2:** Test values of sensitivity and specificity for MLL, PBL, HKL, NMSC, and OBL classification. Specificity and sensitivity values are given for a specific class vs. all others.

	MLL	PBL	HKL	NonMSC	OBL
Specificity (TNR)	0.97	0.90	0.91	0.95	0.93
Sensitivity (TPR)	0.72	0.83	0.61	0.57	0.84

## Data Availability

Data and neural network architectures are available on request.
